# Maternal age and progesterone levels at trigger day as independent predictors of clinical pregnancy in poor ovarian responders undergoing IVF/ICSI: a retrospective analysis

**DOI:** 10.3389/fendo.2025.1593298

**Published:** 2025-06-23

**Authors:** Xingyu Sun, Lijuan He, Ling Liu, Shaohua Wang

**Affiliations:** ^1^ Department of Gynecology, The Affiliated Traditional Chinese Medicine Hospital, Southwest Medical University, Luzhou, Sichuan, China; ^2^ Department of Health Management Center, The Affiliated Hospital, Southwest Medical University, Luzhou, Sichuan, China; ^3^ Department of Reproductive Medicine Center, The Affiliated Hospital, Southwest Medical University, Luzhou, Sichuan, China; ^4^ Department of Pathology, The Affiliated Hospital, Southwest Medical University, Luzhou, China

**Keywords:** poor ovarian response, IVF, ICSI, clinical pregnancy, maternal age, progesterone levels, predictors

## Abstract

**Background:**

Poor ovarian response (POR) during *in vitro* fertilization (IVF) or intracytoplasmic sperm injection (ICSI) significantly compromises clinical pregnancy outcomes. This study evaluated the clinical characteristics and outcomes of IVF/ICSI cycles in poor ovarian responders, focusing specifically on maternal age and progesterone levels at the trigger day as predictors of clinical pregnancy.

**Methods:**

This retrospective study included 652 poor ovarian responders treated with IVF/ICSI between January 2018 and December 2021 at a tertiary fertility center. POR was defined according to the Bologna criteria. Various ovarian stimulation protocols (antagonist, modified natural cycle, short agonist, and long agonist protocols) were employed based on individualized patient assessment. Demographic data, ovarian stimulation details, cycle outcomes, and hormonal levels at trigger day were analyzed. Multivariate logistic regression was performed to identify independent predictors of clinical pregnancy.

**Results:**

Of the 652 patients analyzed, the clinical pregnancy rate was 5.5%. Age, body mass index (BMI), stimulation protocols, and other clinical variables showed no significant differences between pregnant and non-pregnant groups. Multivariate logistic regression analysis identified maternal age (adjusted OR: 1.035; 95% CI: 1.024–1.078; P=0.047) and progesterone levels at the trigger day (adjusted OR: 1.422; 95% CI: 1.380–1.564; P=0.034) as independent predictors of clinical pregnancy.

**Conclusions:**

Maternal age and progesterone levels on the trigger day are independent predictors of clinical pregnancy in poor ovarian responders undergoing IVF/ICSI. Tailoring ovarian stimulation strategies according to these predictive factors may enhance clinical outcomes in this challenging patient population. Further studies exploring advanced techniques and individual patient characteristics are necessary to optimize management strategies for POR patients.

## Introduction

Infertility, defined as the inability to achieve a clinical pregnancy after 12 months or more of regular unprotected sexual intercourse, affects 10-15% of couples worldwide (1). Assisted reproductive technology (ART), including *in vitro* fertilization (IVF) and intracytoplasmic sperm injection (ICSI), has become the primary treatment option for couples struggling with infertility (2). Despite significant advances in ART, poor ovarian response (POR) in IVF/ICSI treatments remains a challenge for reproductive medicine (3).

POR is characterized by a reduced number of developing follicles and a consequent low number of retrieved oocytes following controlled ovarian stimulation (COS) (4). POR occurs in approximately 9-24% of IVF/ICSI cycles and is associated with reduced pregnancy rates and increased cycle cancellation rates (5). Several factors have been proposed to contribute to POR, including advanced maternal age, diminished ovarian reserve, and genetic factors (6, 7). However, the exact etiology of POR remains elusive, making it difficult to develop effective treatment strategies for this challenging patient population.

Various ovarian stimulation protocols have been developed to optimize treatment outcomes in poor ovarian responders, including the antagonist protocol, modified natural cycle (MNC), short agonist protocol, and long agonist protocol (8). Although these protocols have shown some benefits, the optimal stimulation strategy for poor ovarian responders is still a matter of debate (9). Moreover, existing studies on the clinical characteristics and treatment outcomes of poor ovarian responders have reported inconsistent results, and the identification of potential predictors for clinical pregnancy remains an unmet need (10, 11).

Maternal age has long been recognized as an important determinant of fertility and treatment outcomes in ART (12). As women age, the quantity and quality of their oocytes decline, leading to decreased pregnancy rates and increased miscarriage rates (13). Several studies have investigated the association between maternal age and treatment outcomes in poor ovarian responders undergoing IVF/ICSI, but the results have been inconclusive (14, 15). Another potential predictor of clinical pregnancy in poor ovarian responders is the progesterone level on the trigger day. Progesterone, a hormone produced by the corpus luteum, plays a crucial role in the establishment and maintenance of pregnancy (16). Elevated progesterone levels on the day of human chorionic gonadotropin (hCG) administration have been associated with decreased pregnancy rates in IVF/ICSI cycles, possibly due to impaired endometrial receptivity (17). However, the relationship between progesterone levels on the trigger day and treatment outcomes in poor ovarian responders remains unclear (18).

Given the limited and inconsistent evidence on the clinical characteristics and treatment outcomes of poor ovarian responders, as well as the potential predictors of clinical pregnancy, further research is needed to better understand this complex patient population and optimize their treatment strategies. In this retrospective study, we aimed to assess the clinical characteristics and treatment outcomes of poor ovarian responders treated with IVF/ICSI between January 2018 and December 2021, and identify potential predictors for clinical pregnancy, focusing on maternal age and progesterone levels on the trigger day. Understanding the impact of these factors on clinical pregnancy outcomes in poor ovarian responders could have important implications for personalized treatment strategies and improve the chances of pregnancy success for this challenging patient population. Furthermore, this study may help to shed light on the underlying mechanisms contributing to POR and guide future research efforts in the field of reproductive medicine.

## Patients and methods

This retrospective study was conducted in a tertiary fertility center between January 2018 and December 2021. The study population comprised of poor ovarian responders undergoing IVF or ICSI treatments. Poor ovarian response was defined according to the Bologna criteria, which included at least two of the following three features: (1) advanced maternal age (≥ 40 years) or any other risk factor for poor ovarian response, (2) a previous poor ovarian response (≤ 3 oocytes with a conventional stimulation protocol), and (3) an abnormal ovarian reserve test, defined specifically as an antral follicle count (AFC) of less than 5-7, and/or an anti-Mullerian hormone (AMH) level of 0.5-1.1 ng/ml. Patients with missing data, preimplantation genetic testing, or oocyte or embryo cryopreservation were excluded from the study.

Patients were treated with various ovarian stimulation protocols, including the antagonist protocol, modified natural cycle (MNC), short agonist protocol, and long agonist protocol. The choice of protocol was based on the patient’s age, ovarian reserve, and previous response to ovarian stimulation. Data were collected from the electronic medical records and included patients’ demographic characteristics, stimulation details, and treatment outcomes. The primary outcome of interest was clinical pregnancy, defined as the presence of a gestational sac with fetal heartbeat observed on transvaginal ultrasound at 6–7 weeks of gestation.

Statistical analysis was performed as described in the “Statistical Analysis” section. Descriptive statistics were used to summarize the study population’s baseline characteristics, and comparative analyses were conducted to identify differences in outcomes between various subgroups. Multivariate logistic regression analysis was employed to evaluate the potential predictors of clinical pregnancy in poor ovarian responders.

This study was approved by the Institutional Review Board of the fertility center, and the requirement for informed consent was waived due to the retrospective nature of the study. All procedures were performed in accordance with the ethical standards of the institutional research committee and the Helsinki Declaration.

### Statistical analysis

Statistical analysis for the current study was performed using the SPSS software package version 25 (SPSS Inc., Chicago, IL). Continuous variables were presented as means and standard deviations (SD), while categorical variables were presented as numbers and percentages. Differences in variables were statistically analyzed by Student’s t-test, Fisher exact test, and Pearson chi-square test, as appropriate. Normality of variables was assessed via the Shapiro-Wilks test of normality. To further investigate predictors for clinical pregnancy in poor ovarian responders, a multivariate logistic regression analysis was used, controlling for confounding effects that included the patients’ age, BMI, protocol type, day 3 FSH levels, duration of stimulation, total dose of gonadotropins, the number of follicles greater than 15 mm, leading follicle size, E2 levels at the trigger day, trigger type, and the number of retrieved oocytes. Two-sided p-values of < 0.05 were accepted as statistically significant.

## Results

### Baseline characteristics and cycle outcomes of poor ovarian responders undergoing IVF treatment

**Table 1** presents the baseline characteristics and cycle outcomes of poor ovarian responders undergoing IVF treatment (N = 652). The overall mean age of the patients was 37.245 ± 7.4342 years, and the mean BMI was 27.257 ± 5.3277 kg/m². The distribution of the stimulation protocols was as follows: antagonist protocol in 483 cases (74.1%), modified natural cycle (MNC) in 118 cases (18.1%), short agonist protocol in 31 cases (4.8%), and long agonist protocol in 20 cases (3.1%). The mean day 3 FSH levels were 15.852 ± 9.0466 IU/L, and the mean duration of stimulation was 8.9632 ± 3.2088 days. The total dose of gonadotropins used was 4028.3 ± 2232.4 IU. Regarding the cycle outcomes, the mean number of follicles greater than 15 mm was 1.6994 ± 0.79077, and the mean leading follicle size was 18.277 ± 2.1674 mm. The mean E2 level at the trigger day was 1825.1 ± 913.15 pg/mL. The trigger distribution included dual trigger in 206 cases (31.6%) and hCG trigger in 446 cases (68.4%). The mean progesterone levels at the trigger day were 1.5337 ± 1.0309 ng/mL, and the mean number of retrieved oocytes was 1.6626 ± 1.1743. Concerning embryo quality, 269 cycles (41.3%) had one top-quality embryo, 369 cycles (56.6%) had none, and 14 cycles (2.1%) had two top-quality embryos. The pregnancy outcome was positive in 36 cases (5.5%), while 616 cases (94.5%) did not result in pregnancy.

**Table 1:** Baseline characteristics and cycle outcomes of poor ovarian responders undergoing IVF/ICSI treatment.

Characteristics	overall
Age	37.245 ± 7.4342
BMI	27.257 ± 5.3277
Protocol, n (%)	
Antagonist	483 (74.1%)
MNC	118 (18.1%)
Short agonist	31 (4.8%)
Long agonist	20 (3.1%)
Day 3 FSH levels	15.852 ± 9.0466
Duration of stimulation	8.9632 ± 3.2088
Total dose of gonadotropins	4028.3 ± 2232.4
No of follicles >15 mm	1.6994 ± 0.79077
Leading follicle size mm	18.277 ± 2.1674
E2 level at the trigger day	1825.1 ± 913.15
Trigger, n (%)	
Dual trigger	206 (31.6%)
HCG	446 (68.4%)
Progesterone levels at the trigger day	1.5337 ± 1.0309
No of retrieved oocytes	1.6626 ± 1.1743
Top quality embryos, n (%)	
1	269 (41.3%)
0	369 (56.6%)
2	14 (2.1%)
Outcome, n (%)	
Pregnancy	36 (5.5%)
No pregnancy	616 (94.5%)

Values are expressed as mean ± standard deviation or number (percentage). Abbreviations: BMI, body mass index; MNC, modified natural cycle; FSH, follicle-stimulating hormone; E2, estradiol; HCG, human chorionic gonadotropin.

### Comparison of baseline characteristics and cycle outcomes in poor ovarian responders undergoing IVF treatment by age group (40 vs. ≥40 years)

**Table 2** compares the baseline characteristics and cycle outcomes in poor ovarian responders undergoing IVF treatment by age group, specifically those younger than 40 years (n = 230) and those aged 40 years and older (n = 422). The mean age for the younger group was 28.552 ± 6.038 years, while the older group had a mean age of 41.983 ± 1.3567 years (P < 0.001). There was no significant difference in BMI between the two groups (26.768 ± 5.5099 kg/m² in the younger group and 27.524 ± 5.2131 kg/m² in the older group; P = 0.083). There were no significant differences in the distribution of stimulation protocols between the two age groups (P = 0.242). The mean day 3 FSH levels were significantly higher in the older group (16.372 ± 9.4863 IU/L) compared to the younger group (14.9 ± 8.1121 IU/L; P = 0.038). The duration of stimulation was also significantly longer in the older group (9.1682 ± 3.1649 days) compared to the younger group (8.587 ± 3.2614 days; P = 0.027). The total dose of gonadotropins used was significantly higher in the older group (4465.9 ± 2276.2 IU) compared to the younger group (3225.3 ± 1908.4 IU; P < 0.001). However, there were no significant differences in the number of follicles greater than 15 mm, leading follicle size, or E2 levels at the trigger day between the two age groups (P > 0.05 for all comparisons). The distribution of triggers and progesterone levels at the trigger day showed significant differences between the two age groups (P = 0.026). Regarding the number of retrieved oocytes and the distribution of top-quality embryos, there were no significant differences between the two age groups (P > 0.05 for all comparisons). However, the pregnancy outcome showed a significant difference, with the younger group having a higher percentage of pregnancies (3.1%) compared to the older group (2.5%; P = 0.009).

**Table 2:** Comparison of baseline characteristics and cycle outcomes in poor ovarian responders undergoing IVF/ICSI treatment by age group (<40 vs. ≥40 years).

Characteristics	<40	≥40	P value
n	230	422	
Age	28.552 ± 6.038	41.983 ± 1.3567	< 0.001
BMI	26.768 ± 5.5099	27.524 ± 5.2131	0.083
Protocol, n (%)			0.242
Antagonist	171 (26.2%)	312 (47.9%)	
MNC	47 (7.2%)	71 (10.9%)	
Short agonist	7 (1.1%)	24 (3.7%)	
Long agonist	5 (0.8%)	15 (2.3%)	
Day 3 FSH levels	14.9 ± 8.1121	16.372 ± 9.4863	0.038
Duration of stimulation	8.587 ± 3.2614	9.1682 ± 3.1649	0.027
Total dose of gonadotropins	3225.3 ± 1908.4	4465.9 ± 2276.2	< 0.001
No of follicles >15 mm	1.6609 ± 0.84509	1.7204 ± 0.75974	0.374
Leading follicle size mm	18.441 ± 2.1029	18.187 ± 2.1992	0.154
E2 level at the trigger day	1797.6 ± 1052.9	1840.1 ± 828.09	0.597
Trigger, n (%)			0.318
Dual trigger	67 (10.3%)	139 (21.3%)	
HCG	163 (25%)	283 (43.4%)	
Progesterone levels at the trigger day,	1.4188 ± 0.90098	1.5963 ± 1.0911	0.026
No of retrieved oocytes	1.7 ± 1.2543	1.6422 ± 1.1294	0.548
Top quality embryos, n (%)			0.480
1	96 (14.7%)	173 (26.5%)	
0	127 (19.5%)	242 (37.1%)	
2	7 (1.1%)	7 (1.1%)	
Outcome, n (%)			0.009
Pregnancy	20 (3.1%)	16 (2.5%)	
No pregnancy	210 (32.2%)	406 (62.3%)	

Values are expressed as mean ± standard deviation or number (percentage). BMI, body mass index; MNC, modified natural cycle; FSH, follicle-stimulating hormone; E2, estradiol; HCG, human chorionic gonadotropin.

### Comparison of baseline characteristics and cycle outcomes between pregnant and non-pregnant poor ovarian responders undergoing IVF treatment

**Table 3** presents a comparison of the baseline characteristics and cycle outcomes between poor ovarian responders who achieved pregnancy (n = 36) and those who did not (n = 616) following IVF treatment. The mean age was significantly lower in the pregnancy group (35.083 ± 7.2835 years) compared to the no pregnancy group (37.372 ± 7.4292 years; P = 0.043). However, there was no significant difference in BMI between the two groups (26.847 ± 4.6203 kg/m² in the pregnancy group and 27.281 ± 5.3685 kg/m² in the no pregnancy group; P = 0.635). The distribution of stimulation protocols between the two groups did not show any significant differences (P = 0.947). There were also no significant differences in day 3 FSH levels, duration of stimulation, total dose of gonadotropins, the number of follicles greater than 15 mm, leading follicle size, and E2 levels at the trigger day between the two groups (P > 0.05 for all comparisons). Regarding the triggers, there was no significant difference in the distribution between the pregnancy and no pregnancy groups (P = 0.333). The progesterone levels at the trigger day were higher in the no pregnancy group (1.5526 ± 1.0391 ng/mL) compared to the pregnancy group (1.209 ± 0.82418 ng/mL), but this difference did not reach statistical significance (P = 0.052). There was no significant difference in the number of retrieved oocytes between the two groups (1.8611 ± 1.2684 in the pregnancy group and 1.651 ± 1.1687 in the no pregnancy group; P = 0.297). However, the distribution of top-quality embryos showed a significant difference between the two groups (P < 0.001). In the pregnancy group, all patients had at least one top-quality embryo, while in the no pregnancy group, 56.6% had no top-quality embryos.

**Table 3:** Comparison of baseline characteristics and cycle outcomes between pregnant and non-pregnant poor ovarian responders undergoing IVF/ICSI treatment.

Characteristics	Pregnancy	No pregnancy	P value
n	36	616	
Age	35.083 ± 7.2835	37.372 ± 7.4292	0.043
BMI	26.847 ± 4.6203	27.281 ± 5.3685	0.635
Protocol, n (%)			0.947
Antagonist	27 (4.1%)	456 (69.9%)	
MNC	7 (1.1%)	111 (17%)	
Short agonist	1 (0.2%)	30 (4.6%)	
Long agonist	1 (0.2%)	19 (2.9%)	
Day 3 FSH levels	15.779 ± 9.1107	15.857 ± 9.0503	0.960
Duration of stimulation	8.6667 ± 3.5537	8.9805 ± 3.1899	0.569
Total dose of gonadotropins	3998.9 ± 2431.1	4030 ± 2222.4	0.935
No of follicles>15 mm	1.6389 ± 0.68255	1.7029 ± 0.79698	0.637
Leading follicle size mm	18.218 ± 2.2612	18.28 ± 2.1637	0.868
E2 level at the trigger day	1797.4 ± 952.91	1826.7 ± 911.56	0.851
Trigger, n (%)			0.333
Dual trigger	14 (2.1%)	192 (29.4%)	
HCG	22 (3.4%)	424 (65%)	
Progesterone levels at the trigger day	1.209 ± 0.82418	1.5526 ± 1.0391	0.052
No of retrieved oocytes	1.8611 ± 1.2684	1.651 ± 1.1687	0.297
Top quality embryos, n (%)			< 0.001
1	35 (5.4%)	234 (35.9%)	
0	0 (0%)	369 (56.6%)	
2	1 (0.2%)	13 (2%)	

Values are expressed as mean ± standard deviation or number (percentage). BMI, body mass index; MNC, modified natural cycle; FSH, follicle-stimulating hormone; E2, estradiol; HCG, human chorionic gonadotropin.

### Univariate and multivariate logistic regression analysis of factors associated with pregnancy outcomes in poor ovarian responders undergoing IVF cycles

**Table 4** displays the results of univariate and multivariate logistic regression analyses of factors potentially associated with clinical pregnancy in poor ovarian responders (total N = 652). In the univariate analysis, age was found to be significantly associated with clinical pregnancy, with an odds ratio (OR) of 1.037 (95% CI: 1.006 - 1.039; P = 0.026). However, BMI, stimulation protocol, day 3 FSH levels, duration of stimulation, total dose of gonadotropins, the number of follicles greater than 15 mm, leading follicle size, E2 levels at the trigger day, trigger type, and the number of retrieved oocytes were not significantly associated with clinical pregnancy (P > 0.05 for all comparisons). In the multivariate analysis, age remained significantly associated with clinical pregnancy, with an adjusted OR of 1.035 (95% CI: 1.024 - 1.078; P = 0.047). Progesterone levels at the trigger day also showed a significant association with clinical pregnancy, with an adjusted OR of 1.422 (95% CI: 1.380 - 1.564; P = 0.034). Other factors included in the analysis did not show significant associations with clinical pregnancy in the multivariate model.

**Table 4:** Univariate and multivariate logistic regression analysis of factors associated with pregnancy outcomes in poor ovarian responders undergoing IVF/ICSI cycles.

Characteristics	Total (N)	Univariate analysis		Multivariate analysis	
		Odds Ratio (95% CI)	P value	Odds Ratio (95% CI)	P value
Age	652	1.037 (1.006 - 1.039)	0.026	1.035 (1.024 - 1.078)	**0.047**
BMI	652	1.015 (0.953 - 1.082)	0.634		
Protocol	652				
Antagonist	483	Reference			
MNC	118	0.939 (0.399 - 2.212)	0.885		
Short agonist	31	1.776 (0.233 - 13.523)	0.579		
Long agonist	20	1.125 (0.145 - 8.722)	0.910		
Day 3 FSH levels	652	1.001 (0.964 - 1.039)	0.960		
Duration of stimulation	652	1.031 (0.929 - 1.143)	0.568		
Total dose of gonadotropins	652	1.000 (1.000 - 1.000)	0.935		
No of follicles >15 mm	652	1.108 (0.723 - 1.698)	0.637		
Leading follicle size mm	652	1.013 (0.867 - 1.184)	0.868		
E2 level at the trigger day	652	1.000 (1.000 - 1.000)	0.851		
Trigger	652				
Dual trigger	206	Reference			
hCG	446	1.405 (0.704 - 2.806)	0.335		
Progesterone levels at the trigger day	652	1.437 (1.295 - 1.675)	0.023	1.422 (1.380 - 1.564)	**0.034**
No of retrieved oocytes	652	0.867 (0.663 - 1.134)	0.297		

BMI, body mass index; MNC, modified natural cycle; FSH, follicle-stimulating hormone; E2, estradiol; hCG, human chorionic gonadotropin Bold values indicate statistically significant results (P < 0.05).

### Receiver operating characteristic analysis of age and progesterone levels at the trigger day, and the model for predicting clinical pregnancy in poor ovarian responders

**Figure 1:**
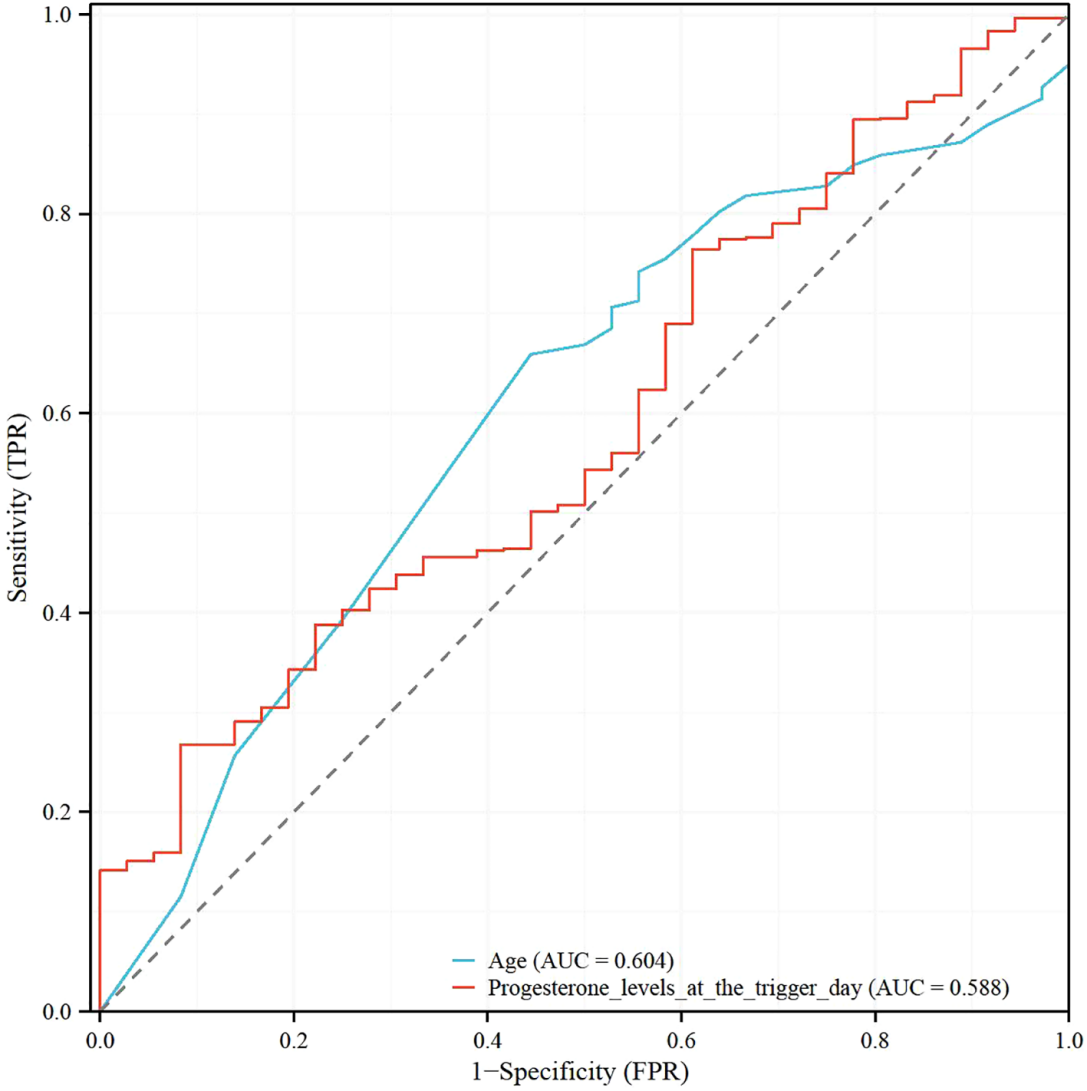
Receiver operating characteristic (ROC) analysis of age and progesterone levels at the trigger day in poor ovarian responders.

**Figure 2:**
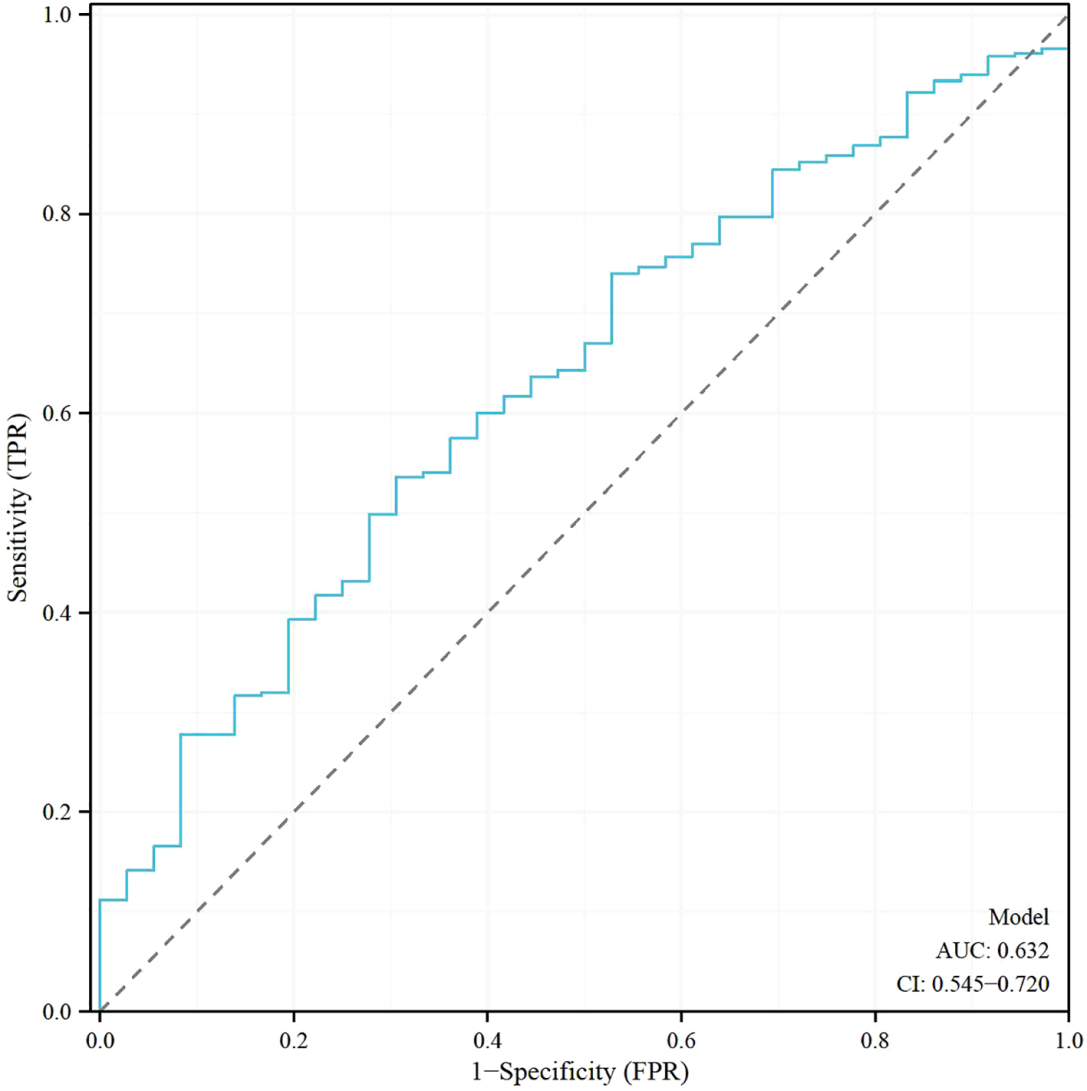
Receiver operating characteristic (ROC) Analysis of the model for predicting clinical pregnancy in poor ovarian responders.

**Figure 3:**
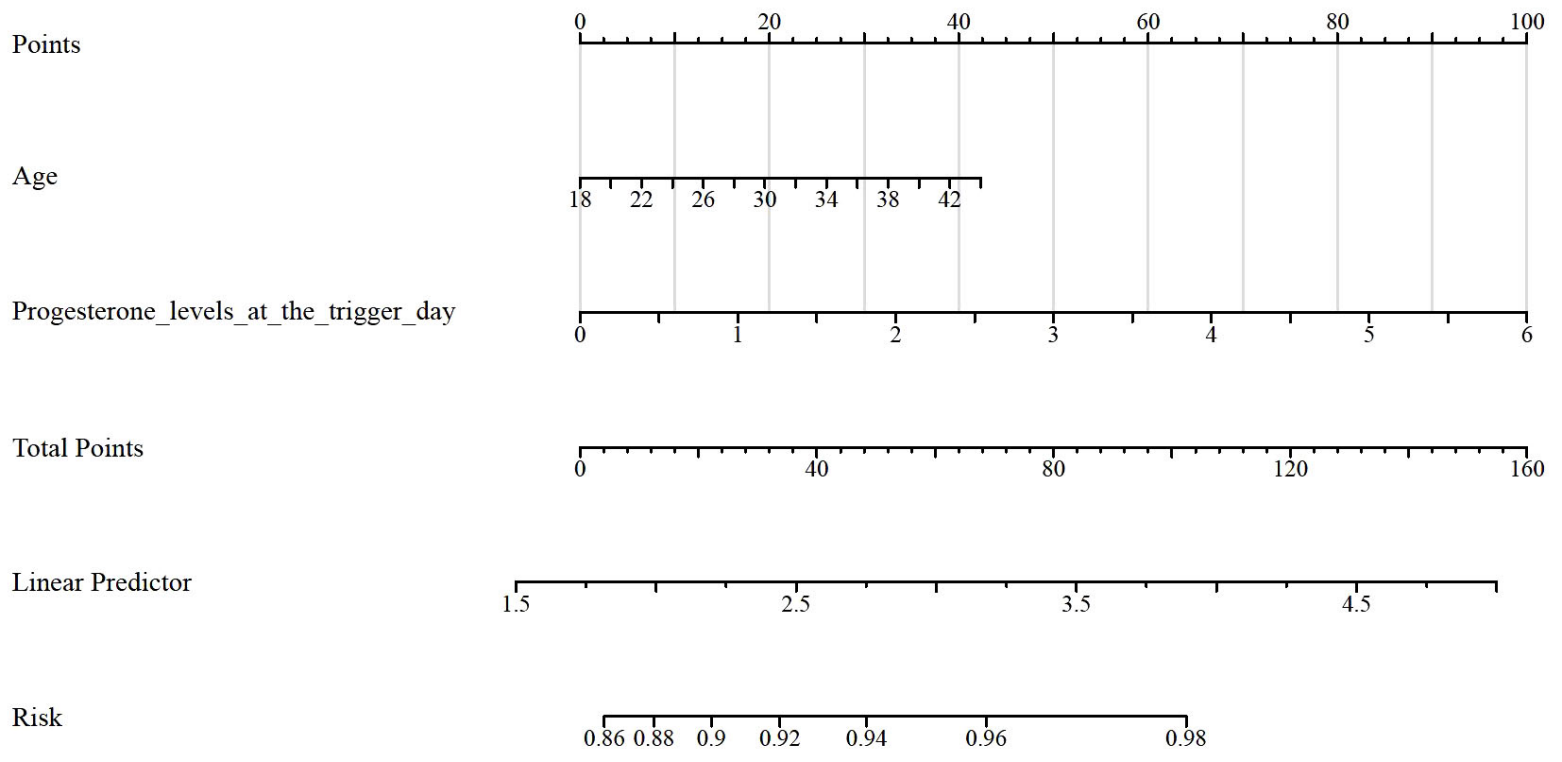
Nomogram that can predict clinical pregnancy in poor ovarian responders.

**Table 5** shows the results of the Receiver Operating Characteristic (ROC) analysis for age, progesterone levels at the trigger day, and the combined model, in predicting clinical pregnancy among poor ovarian responders. The Area Under the Curve (AUC) for age was 0.604, with a 95% confidence interval (CI) ranging from 0.512 to 0.696, indicating a moderate predictive accuracy. The optimal cut-off value for age was found to be 39.5 years, with a specificity of 55.56% and a sensitivity of 65.91%. Progesterone levels at the trigger day presented an AUC of 0.588 (95% CI: 0.497 - 0.679), suggesting slightly less predictive accuracy than age. The optimal cut-off value for progesterone levels was determined to be 2.1756 ng/mL. This threshold, while offering a high sensitivity of 91.67%, was associated with a comparatively low specificity of 26.79%. The combined model, integrating both age and progesterone levels at the trigger day, offered an increased AUC of 0.632 (95% CI: 0.545 - 0.720). This illustrates the improved predictive power of the model, with the specificity and sensitivity being 69.44% and 53.57% respectively at an optimal cut-off value of 2.8728. More results can be found in **Figures 1**, **2**. These findings underscore the potential utility of age and progesterone levels on the trigger day as predictors for clinical pregnancy in poor ovarian responders. Moreover, they highlight the increased predictive power achieved by considering both variables concurrently in a combined model. Furthermore, a nomogram was developed based on maternal age and progesterone levels at the trigger day to visually aid in predicting clinical pregnancy in poor ovarian responders (**Figure 3**).

**Table 5:** Receiver operating characteristic (ROC) analysis of age and progesterone levels at the trigger day, and the model for predicting clinical pregnancy in poor ovarian responders.

Parameters	AUC	95% CI	cut-off value	Specificity (%)	Sensitivity (%)
Age	0.604	0.512 - 0.696	39.5	0.55556	0.65909
Progesterone levels at the trigger day	0.588	0.497 - 0.679	2.1756	0.26786	0.91667
Model	0.632	0.545 - 0.720	2.8728	0.69444	0.53571

AUC stands for Area Under the Curve, which is a measure of the predictive accuracy of the model. The 95% CI refers to the 95% Confidence Interval. Cut-off value is the threshold at which the parameter’s effect on the outcome is maximized. Specificity refers to the proportion of true negative results in the population, and Sensitivity refers to the proportion of true positive results. The Model refers to a combined analysis of age and progesterone levels at the trigger day.

## Discussion

In this comprehensive retrospective analysis, we reviewed the clinical characteristics and treatment outcomes of 652 poor ovarian responders undergoing IVF/ICSI treatment. Our findings have led us to conclude that maternal age and progesterone levels on the day of the trigger emerged as independent predictors of clinical pregnancy in poor ovarian responders.

The overall pregnancy rate registered in our study was 5.5%, aligning with previous studies revealing decreased pregnancy rates in poor ovarian responders relative to normal responders (4, 5). This marked reduction underscores the need for a more profound understanding of this subgroup of patients and the requirement to optimize their treatment strategies.

The significant correlation of age with clinical pregnancy was evident in both univariate and multivariate analyses. This observation corroborates previous studies stating that advanced maternal age is a potent determinant affecting the success rates in IVF/ICSI cycles (9, 19). The age-associated decline in ovarian function and oocyte quality has been thoroughly investigated and is known to adversely impact IVF/ICSI success rates (6, 13). Some studies indicate that inositol supplementation might enhance oocyte quality and affect subsequent clinical outcomes (20). These findings underline the importance of considering maternal age and oocyte quality while devising personalized treatment plans for poor ovarian responders.

Complementing the age factor, our analysis revealed progesterone levels on the trigger day to be significantly associated with clinical pregnancy outcomes. This supports earlier research hinting that elevated progesterone levels might compromise endometrial receptivity and subsequently, pregnancy rates in IVF/ICSI cycles (21, 22). The specific mechanism responsible for this association is yet to be unraveled, thus necessitating further investigations to elucidate the role of progesterone levels on the trigger day in treatment outcomes for poor ovarian responders.

Contrary to expectations, no significant differences in pregnancy rates were observed among different stimulation protocols employed in our study population. This observation aligns with certain earlier studies reporting no notable differences in treatment outcomes among antagonist, MNC, short agonist, and long agonist protocols in poor ovarian responders (23, 24). Yet, some studies reported contrasting results, thereby fueling ongoing debates about the optimal stimulation strategy for poor ovarian responders (3, 5).

Our study has several limitations. First, its retrospective nature may introduce selection bias and limit the generalizability of our findings. Second, the relatively small sample size of pregnant patients might have affected the statistical power of the study. Third, our study did not consider potential confounding factors, such as genetic variables, lifestyle factors, the quality of the IVF laboratory, and thyroid dysfunction (25). Furthermore, another consideration is the psychological implications of participating in an IVF program, a stressor that significantly impacts couples on their fertility journey (26). Comprehensive care should, therefore, be multidimensional, encompassing both physical and psychological health. For patients undergoing cancer treatments like chemotherapy or radiotherapy, fertility preservation through methods such as oocyte vitrification is recommended (27). Comparing the safety of open versus closed vitrification systems merits further research in light of contrasting studies in the literature (28). Additionally, our study focused on clinical pregnancy as the primary outcome, whereas the live birth rate, a critical measure of success in IVF/ICSI treatments, was not fully assessed due to constraints on available data. Future studies should regard live birth rate as a key outcome to provide a more complete understanding of the factors affecting IVF/ICSI success in poor ovarian responders.

The potential long-term impact of IVF on neonatal outcomes also warrants consideration, including neuro-psycho-motor implications for children conceived through assisted reproductive techniques (29, 30). Research has revealed a potential increased risk of congenital heart diseases in children born through assisted reproductive techniques (20). The advent of non-invasive prenatal diagnostic techniques, such as cell-free fetal DNA, could facilitate early identification of chromosomopathies and pediatric monogenic diseases in children conceived via these treatments (31). In addition, new technologies like artificial intelligence could potentially enhance assisted reproduction outcomes, opening new avenues in oocyte or embryo selection, error reduction, and optimization of treatment strategies (32). Although our study did not directly explore this aspect, the potential of these technologies illustrates the ongoing evolution of the field and the importance of maintaining a broad perspective when considering strategies to improve clinical pregnancy outcomes in poor ovarian responders. Moreover, the emerging field of ovarian tissue cryopreservation and transplantation presents new potential treatment avenues for poor ovarian responders (33). Given the challenges associated with treating this patient group, the potential role of these techniques merits further investigation.

We also recognize the need to consider the broader implications of these treatments on the long-term health of children conceived through IVF. As mentioned before, there exists a potential increased risk of congenital heart diseases in children born through assisted reproductive techniques (20). Finally, our study underscores the criticality of considering maternal age and progesterone levels on the trigger day in the context of clinical pregnancy outcomes in poor ovarian responders undergoing IVF/ICSI treatment. It is envisaged that treatment strategies tailored to these factors may contribute to improved outcomes for this specific patient population. Nevertheless, it is of essence that further large-scale, prospective studies are undertaken to validate our findings and explore additional predictors of clinical pregnancy in poor ovarian responders.

In conclusion, while our study contributes to a greater understanding of factors influencing IVF/ICSI success rates in poor ovarian responders, it also highlights the need for comprehensive, multi-faceted strategies to improve outcomes. The need for a broader perspective in patient management, encompassing not only biological but also psychological considerations, is emphasized. Future research should take into account the potential of novel technologies and methods, as well as the long-term implications of IVF treatments on the health and wellbeing of resulting children.

## Data Availability

The original contributions presented in the study are included in the article/supplementary material. Further inquiries can be directed to the corresponding author.
